# SMEA-YOLOv8n: A Sheep Facial Expression Recognition Method Based on an Improved YOLOv8n Model

**DOI:** 10.3390/ani14233415

**Published:** 2024-11-26

**Authors:** Wenbo Yu, Xiang Yang, Yongqi Liu, Chuanzhong Xuan, Ruoya Xie, Chuanjiu Wang

**Affiliations:** 1 College of Mechanical and Electrical Engineering, Inner Mongolia Agricultural University, Hohhot 010018, China; sa0726yx@163.com (X.Y.); liuyongqi0222@163.com (Y.L.); xcz@imau.edu.cn (C.X.); xie2014030130@163.com (R.X.); 15023171040@163.com (C.W.); 2 Inner Mongolia Engineering Research Center for Intelligent Facilities in Prataculture and Livestock Breeding, Hohhot 010018, China

**Keywords:** expression recognition, YOLOv8n, SimAM, MobileViTAttention, EfficiCIoU

## Abstract

Sheep facial expressions are valuable indicators of their pain levels, playing a critical role in monitoring their health and welfare. In response to challenges such as missed detections, false positives, and low recognition accuracy in sheep facial expression recognition, this paper introduces an enhanced algorithm based on YOLOv8n, referred to as SimAM-MobileViTAttention-EfficiCIoU-AA2_SPPF-YOLOv8n (SMEA-YOLOv8n). Firstly, the proposed method integrates the parameter-free Similarity-Aware Attention Mechanism (SimAM) and MobileViTAttention modules into the CSP Bottleneck with 2 Convolutions(C2f) module of the neck network, aiming to enhance the model’s feature representation and fusion capabilities in complex environments while mitigating the interference of irrelevant background features. Additionally, the EfficiCIoU loss function replaces the original Complete IoU(CIoU) loss function, thereby improving bounding box localization accuracy and accelerating model convergence. Furthermore, the Spatial Pyramid Pooling-Fast (SPPF) module in the backbone network is refined with the addition of two global average pooling layers, strengthening the extraction of sheep facial expression features and bolstering the model’s core feature fusion capacity. Experimental results reveal that the proposed method achieves a mAP@0.5 of 92.5%, a Recall of 91%, a Precision of 86%, and an F1-score of 88.0%, reflecting improvements of 4.5%, 9.1%, 2.8%, and 6.0%, respectively, compared to the baseline model. Notably, the mAP@0.5 for normal and abnormal sheep facial expressions increased by 3.7% and 5.3%, respectively, demonstrating the method’s effectiveness in enhancing recognition accuracy under complex environmental conditions.

## Simple Summary

Sheep often show signs of pain through their facial expressions, making these cues essential for monitoring their health and well-being. Quickly and accurately detecting these expressions is vital for effective pain management and preventing the spread of disease. However, existing detection methods can be slow, inaccurate, or unreliable, especially in challenging conditions like poor lighting or when sheep are partially hidden. To address these issues, we developed a new computer algorithm that automatically recognizes sheep facial expressions associated with pain. Our approach improves upon existing models by more precisely focusing on key facial features and filtering out irrelevant background information. In our tests, the algorithm showed significant improvements in accuracy, more reliably identifying normal and abnormal expressions compared to previous methods. This advancement allows farmers and veterinarians to monitor sheep health more effectively in real time, enabling quicker interventions when animals are in discomfort. Overall, our work provides a practical tool to enhance animal welfare by ensuring sheep receive timely and appropriate care.

## 1. Introduction

Sheep facial expressions are valuable indicators of their pain levels, playing a critical role in monitoring their health and welfare. Accurate pain assessment is essential for effective pain management and welfare evaluation, enabling the timely identification of abnormal conditions and reducing the risk of disease spread from delayed detection. Among pain indicators, facial expressions are considered one of the most reliable. When sheep are ill, they often show signs such as facial distress, rapid breathing, and reduced appetite (YAO et al., 2021) [1]. As a non-invasive and easily observable method, facial expression analysis provides an efficient means for pain assessment, offering a practical tool for early distress detection by farmers and veterinarians. The early identification of pain is crucial for minimizing suffering, enhancing animal welfare, and improving productivity in farming operations. Moreover, using facial expressions for pain detection allows for prompt intervention, which is critical for preventing the worsening of health conditions and ensuring appropriate care. Therefore, accurately assessing sheep pain through facial expressions is fundamental to both their immediate health and long-term welfare.

McLennan et al. introduced the standardized Sheep Pain Facial Expression Scale (SPFES), using footrot and mastitis as models for pain assessment, which assesses pain in specific areas of the face, such as the ears, nostrils, and eyes [2]. Among the scoring methods, a total score exceeding 1.5—derived from assessing the eye area, cheeks, ears, lips, and nose—has been identified as a critical threshold for indicating the presence of pain. This threshold was established through extensive data validation, ensuring an optimal balance between sensitivity and specificity. Specifically, in conditions such as footrot and mastitis, a score of 1.5 demonstrated high sensitivity while maintaining acceptable specificity, effectively distinguishing pain-related facial expressions with minimal misclassification. The 1.5 threshold captures consistent and significant facial changes associated with pain, such as orbital tightening, cheek muscle contraction, altered ear positions, and variations in lip and nostril profiles. These changes indicate clear deviations from a relaxed facial state, providing reliable indicators of pain. Despite its proven reliability in controlled environments, the manual scoring system presents limitations for large-scale farming due to its labor-intensive nature, highlighting the need for more efficient and automated pain detection methods. To overcome these issues, Lu et al. developed a machine learning-based method for recognizing sheep facial expressions according to SPFES [3]. This approach uses Histograms of Oriented Gradients (HOG) to describe facial features and a Support Vector Machine (SVM) to classify pain levels. Despite its effectiveness, the method is limited to frontal facial expressions and does not account for variations caused by different postures. Pessanha et al. advanced a method that combines HOG-based appearance descriptors with geometric posture features to create a binary SVM classifier for pain detection [4]. However, its reliance on manual feature extraction limits its ability to capture the complex relationships in sheep facial expressions. In contrast, deep learning methods offer advantages by automatically extracting features through multi-layer nonlinear transformations, which are better suited for complex expression recognition tasks. Noor et al. improved classification accuracy by applying transfer learning and fine-tuning to VGG16 and ResNet models, enabling differentiation between normal and abnormal expressions [5]. However, this approach does not effectively filter out irrelevant features in complex environments unrelated to pain. Han et al. incorporated a spatial transformer network into VGGNet to focus on key areas indicating pain in sheep, resulting in the Spatial Transformer Visual Geometry Group Network (STVGGNet) [6]. By leveraging transfer learning and fine-tuning, this model effectively classifies facial expressions as painful or pain-free. However, the model’s computational complexity and large parameter size present challenges for deployment in resource-constrained environments.

The accuracy of the sheep facial expression recognition task is often affected by factors like poor lighting and partial occlusions in the environment. Therefore, it is crucial to eliminate features unrelated to pain and filter out irrelevant information from sheep facial expressions. However, existing models and algorithms have struggled to meet these demands, leading to less-than-ideal performance. To address these challenges, this paper proposes SMEA-YOLOv8n, an improved sheep facial expression recognition algorithm based on the YOLOv8n model, chosen for its speed and efficiency. The key contributions of this work are (1) the fusion of the SimAM module into the first and third C2f modules in the neck network, which connects to the Detect head, and the integration of the MobileViTAttention module into the second C2f module; (2) the introduction of an EfficiCIoU loss function that optimizes bounding box predictions by combining the advantages of CIoU and EIoU loss functions and (3) the refinement of the SPPF module in the backbone network, transforming it into AA2_SPPF by adding two global average pooling layers to the original design.

The remainder of this paper is structured as follows. Section 2 describes the materials and methods, beginning with an overview of the sheep facial expression dataset collection process, along with the data preprocessing procedures. This is followed by a detailed explanation of the individual modules within the improved SMEA-YOLOv8n model. Section 3 outlines the experimental setup and presents the performance evaluation of the improved SMEA-YOLOv8n model using metrics such as mAP and Recall. Section 4 provides an analysis and discussion of the experimental results, critically assessing the effectiveness and limitations of the proposed method while suggesting directions for future research. Finally, Section 5 summarizes the main research findings and addresses the research questions.

## 2. Materials and Methods

### 2.1. Experimental Data

#### 2.1.1. Collection of Sheep Facial Expression Data and Image Features

**Figure 1:**
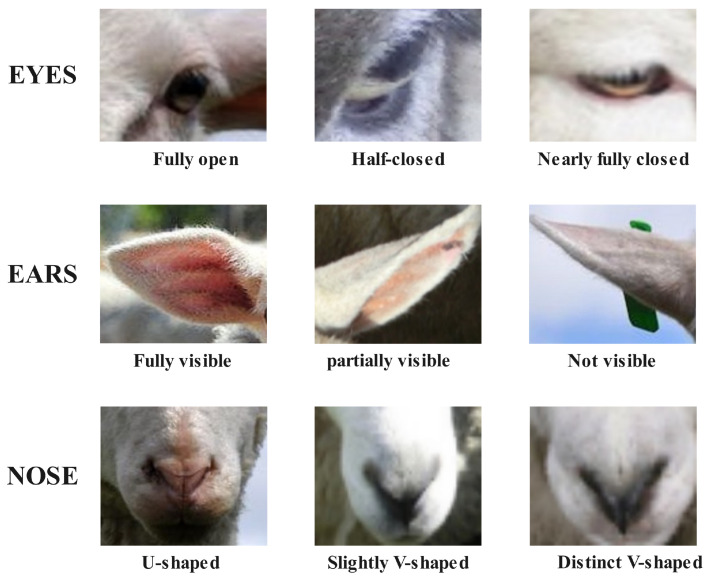
Images of Eyes, Ears, and Nose compliant with SPFES.

The Sheep Pain Facial Expression Scale (SPFES) is a widely recognized method for assessing pain in sheep based on facial expressions. This standard method explained in Table 1, evaluates the degree of contraction in the eyes, the position of the ears, and the shape of the nostrils to assess the possible presence of pain in each region [2]. As shown in Figure 1, the eyes are scored as 0 when fully open (indicating no pain), 1 when half-closed (indicating possible pain), and 2 when nearly fully closed (indicating a higher likelihood of pain). Similarly, for the ears, a visible ear shape is scored as 0, a partially visible ear as 1, and an entirely hidden ear as 2. For the nostrils, a U-shaped nostril is scored as 0 (indicating no pain), a slightly V-shaped nostril as 1 (indicating possible pain), and a pronounced V-shape as 2 (indicating a high likelihood of pain). The scores for the eyes, ears, and nostrils are then summed, and if the total score exceeds 1.5, the sheep is considered to be in pain, while a score of 1.5 or lower indicates a normal condition [2].

It is important to note that while each individual facial feature is scored as an integer (0, 1, or 2), the total score can occasionally be a non-integer value, such as 1.5. This occurs when trained observers assign intermediate scores (e.g., 0.5) to features with ambiguous or borderline characteristics. For example, the eyes may appear slightly more contracted than “Partially present (1)” but not fully meet the criteria for “Present (2)”, leading to a score of 1.5 when summed with other features. Such intermediate scoring improves the granularity and sensitivity of pain detection, as previously demonstrated in similar SPFES-based studies [2]. Noor et al. assembled a dataset comprising 2350 sheep facial images, scored according to the SPFES criteria and sourced from platforms such as Pixabay and ImageNet [5]. While the images were of high resolution to facilitate the accurate identification of facial features, the SPFES standard itself does not prescribe any specific resolution requirements. The dataset is publicly accessible at https://data.mendeley.com/datasets/y5sm4smnfr/3. These images were categorized into two groups as follows: 1407 normal and 943 abnormal, following the SPFES criteria detailed in previous studies [2].

The dataset utilized in this research consists of two parts: a publicly available dataset of sheep facial expressions and a custom-built dataset. The public dataset includes that released by Noor et al. [5] (available at https://data.mendeley.com/datasets/y5sm4smnfr/3), supplemented with an additional 426 images obtained from publicly available sources such as Pixabay (https://pixabay.com) accessed on 13 April 2024 and VEER (https://veer.com) accessed on 13 April 2024. The custom dataset was collected at the Beiqi Smart Breeding Demonstration Base, operated by Inner Mongolia Beiqi Pharmaceutical and Equipment Co., Ltd. (Hohhot, Inner Mongolia Autonomous Region, China). High-resolution images were captured using Hikvision network infrared night-vision surveillance cameras (DC-2CD3T56FWDV2-I5), placed at a height of 10–20 cm above the average shoulder level of sheep and positioned 1–2 m outside the pen. Video was recorded continuously under various conditions, including sunny and overcast days, to ensure 24-h monitoring. The video data were transmitted via Ethernet and Wi-Fi to a remote server. Adobe Premiere Pro 2019 was used to cut irrelevant footage, while Python’s OpenCV library extracted frames at a frequency of 50 frames per second, yielding 325 high-quality images in PNG format. The dataset represents a range of sheep breeds and developmental stages, covering diverse environmental conditions. In this study, the sheep facial expression dataset is divided into two parts: one part consists of normal sheep faces without signs of pain, while the other part includes abnormal faces showing varying levels of pain.

#### 2.1.2. Data Preprocessing

**Figure 2:**
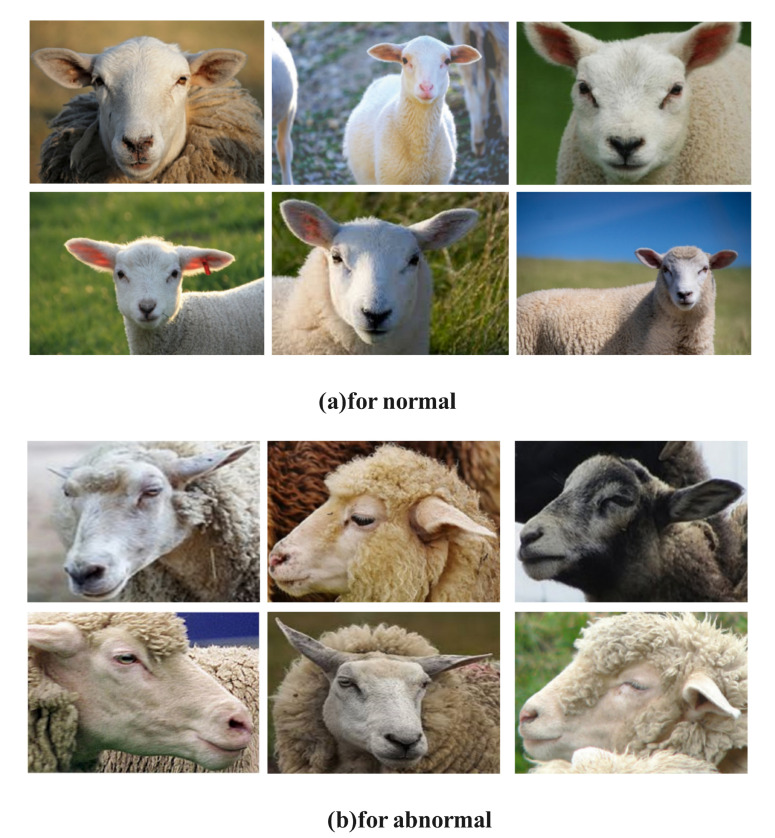
A selection of sheep facial expression images (**a**) for normal (**b**) for abnormal.

First, unclear and duplicate blurry sheep facial expression images from the publicly available Noor dataset were excluded, resulting in 938 normal and 154 abnormal sheep facial expression images. Next, five trained professionals, who were well-versed in the SPFES standard, evaluated and classified an additional 751 sheep facial expression images. These images were sourced from the Pixabay and VEER websites, as well as a custom dataset collected from a ranch. To ensure reliable and consistent annotation, the five professionals underwent rigorous training on the SPFES scoring system, which included detailed discussions of the facial feature evaluation criteria, such as Eye Constriction Status, Ear Rotation Degree, and Nostril Configuration. The training process also involved reviewing previously annotated datasets and performing calibration exercises to ensure inter-observer consistency. Each image was independently assessed by all five professionals, and the final classification for each image was determined by calculating the average score assigned by the five annotators. Discrepancies and borderline cases were resolved through consensus discussions, ensuring objectivity and minimizing individual bias. The classification results are shown in Table 2. Of these 751 images, 62 were identified as abnormal, with scores exceeding 1.5, while 689 were classified as normal, with scores below 1.5. The sheep facial expression dataset used in this study consists of 1843 images, including 1627 normal and 216 abnormal facial expression images. Abnormal and normal sheep facial expressions were classified based on the SPFES standard. However, it is important to note that no specific diagnosable painful conditions (e.g., foot rot or mastitis) were used to further calibrate the abnormal group. Figure 2 provides examples of sheep facial expressions from the dataset.

**Figure 3:**
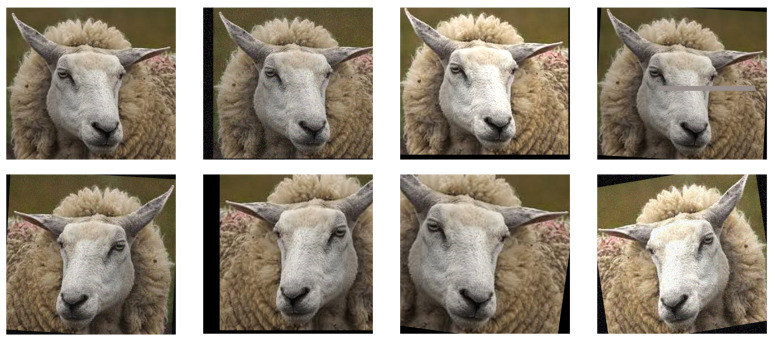
Augmentation techniques for abnormal sheep facial expression image data.

**Figure 4:**
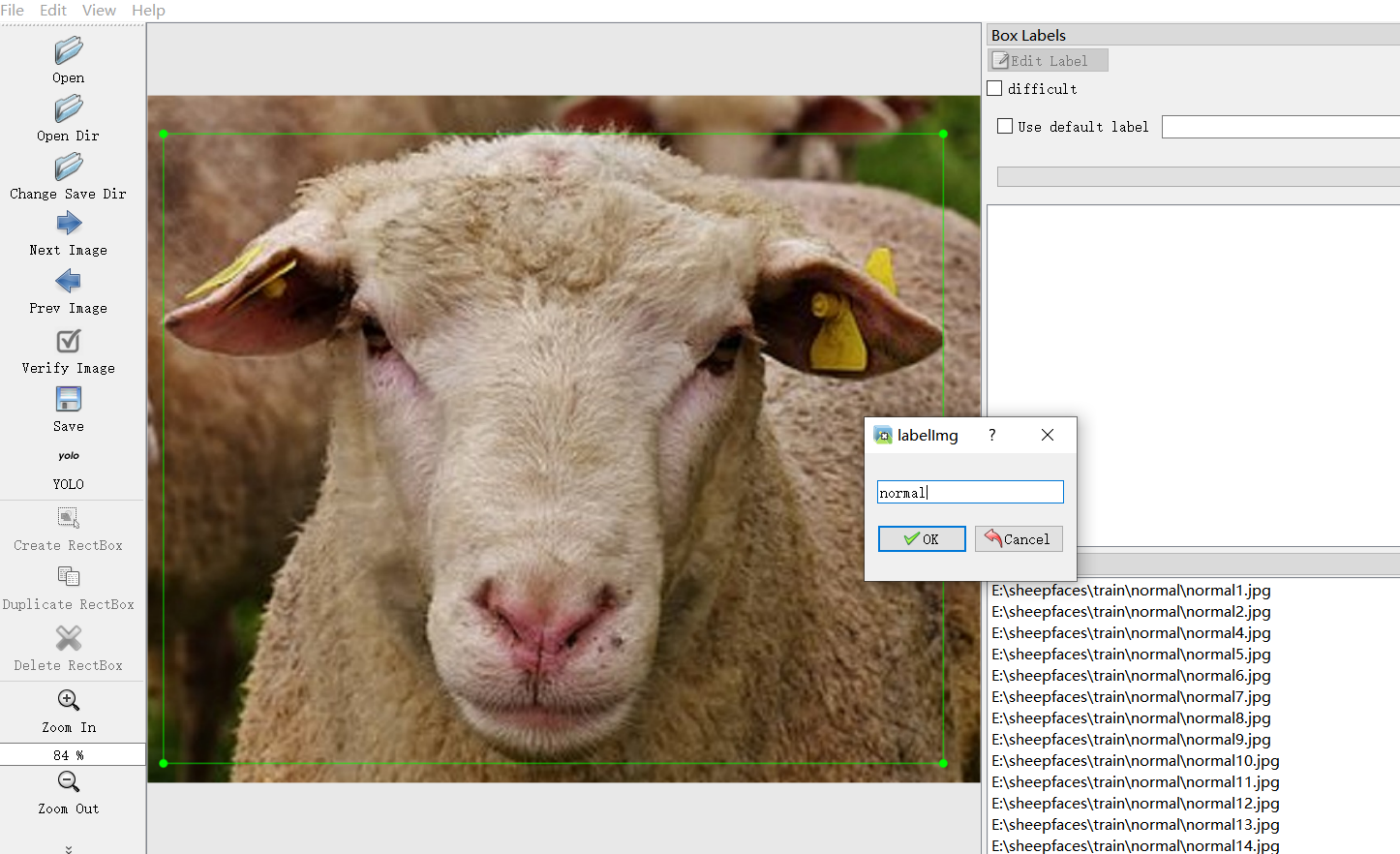
Annotation of sheep facial expression dataset.

To mitigate the limited availability of sheep facial expression images, which can contribute to model overfitting, this study employs a diverse range of data augmentation techniques—such as rotation, translation, cropping, flipping, mosaic augmentation, brightness and contrast adjustments, and noise injection—in randomized combinations to expand the dataset by a factor of 5. This comprehensive augmentation strategy is designed to improve model robustness and generalization, expediting training and enhancing recognition accuracy. Initially, random rotation within a range of [−10°, 10°] and translation up to 10% of the image width and height was applied with a 50% probability to simulate variations in facial expressions at different angles. To further support the model’s ability to recognize partially occluded or non-standard viewing angles, random cropping and flipping were executed with a 50% probability, while mosaic augmentation was applied with a 20% probability. Additionally, brightness and contrast adjustments were randomly introduced to improve the model’s resilience to different lighting conditions, with a probability of 30%. Finally, to increase robustness in noisy environments, Gaussian, salt-and-pepper, and speckle noise were applied randomly to mimic environmental noise during image acquisition. The Gaussian noise had a standard deviation of 0.02, salt-and-pepper noise had a density of 0.01, and speckle noise had an intensity of 0.02, each with a 30% probability. As shown in Figure 3, the augmented dataset ultimately comprised 9215 images of sheep facial expressions, including 8135 normal expressions and 1080 abnormal expressions. This dataset was divided into training, validation, and test sets in a 7:2:1 ratio, containing 6388, 1825, and 1002 images, respectively. To ensure accurate sheep facial expression classification, all images were labeled using the LabelImg tool, as shown in Figure 4, with normal expressions marked as “normal” and abnormal expressions as “abnormal”.

### 2.2. SMEA-YOLOv8n Model for Sheep Facial Expression Recognition

To effectively filter out features in sheep facial expressions that are unrelated to pain while suppressing irrelevant information, the SMEA-YOLOv8n model incorporates several key components as follows: the SimAM attention module, the MobileViTAttention module, the EfficiCIoU-Loss module, and the AA2_SPPF module. As demonstrated by Jiang et al. [7], the parameter-free SimAM module, used to replace the SENet attention mechanism in the MobileNetV3 network, significantly reduced network parameters while boosting the model’s ability to extract relevant features. This led to enhanced accuracy in facial expression recognition. The SMEA-YOLOv8n model integrates the SimAM module with the three C2f modules in the neck network, which are linked to the Detect network, promoting better feature fusion during sheep facial expression analysis and improving the detection of smaller targets. In addition, some of the C2f modules are combined with the MobileViTAttention module. To further improve training efficiency and accelerate convergence, the CIoU loss function was replaced by the EfficiCIoU loss function, which increases the model’s robustness. Finally, the improved AA2_SPPF module captures global contextual information of sheep facial expressions more effectively and helps reduce the impact of varying scales.

**Figure 5:**
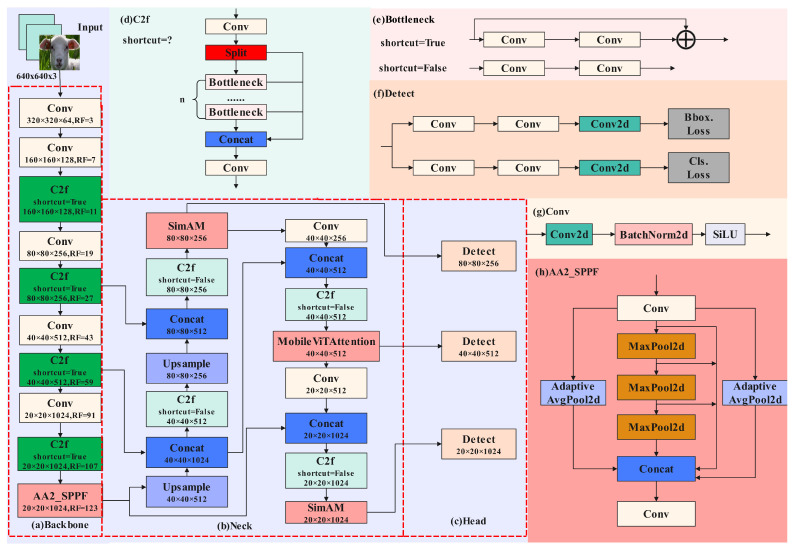
Architecture of the SMEA-YOLOv8n model for sheep facial expression recognition.

To achieve the accurate detection of pain-related facial expressions in sheep while filtering out irrelevant information, the SMEA-YOLOv8n model integrates a set of critical components, each serving distinct roles in feature extraction, fusion, and multi-scale detection, as illustrated in Figure 5. The model architecture comprises (a) Backbone, (b) Neck, (c) Head, (d) C2f module, (e) Bottleneck module, (f) Detect module, (g) Conv module, and (h) AA2_SPPF module, each contributing to the overall performance as follows:

(a) Backbone: This section handles the extraction of fundamental features from the input images and serves as the core feature extraction component. The Backbone consists of several convolutional layers and C2f modules, which progressively capture multi-scale features. C2f modules configured with shortcut = True allow effective information flow, preserving feature continuity and enhancing computational efficiency. The Backbone gradually increases the feature depth, enabling a rich representation for subsequent processing stages.
(b) Neck: This component facilitates multi-scale feature fusion and includes the SimAM and MobileViTAttention modules. SimAM is a parameter-free attention mechanism designed to replace the traditional SENet by emphasizing key regions directly in the feature map. This approach reduces the parameter count while improving feature relevance. The MobileViTAttention module further strengthens the model’s capacity to capture fine-grained features, which is especially beneficial for detecting smaller expressions, enhancing the model’s robustness in challenging scenarios.
(c) Head: The Head component consists of multiple Detect layers which output the final classification and localization results. Each Detect layer processes features at various scales, optimizing detection across different resolutions. The inclusion of classification loss (Cls. Loss) and bounding box loss (Bbox. Loss) in the Head ensures precise recognition and localization of facial expressions.
(d) C2f Module: The C2f module is crucial for efficient feature extraction, utilizing split, bottleneck, and concat operations to manage feature flows. Depending on the shortcut setting, it either maintains feature continuity for shallow layers (shortcut = True) or focuses on deep feature abstraction (shortcut = False), adapting to the complexity of facial expressions.
(e) Bottleneck Module: Located within the Backbone and Neck, the Bottleneck module utilizes a series of convolutions, with and without shortcut connections. When configured with shortcut = True, it preserves feature integrity; when shortcut = False, it emphasizes deeper feature processing, enhancing the model’s adaptability to complex expression patterns.
(f) Detect Module: The Detect module is responsible for classification and localization tasks. Each Detect layer is designed to process inputs from distinct feature scales, enabling the model to handle expressions of varying sizes. It employs both bounding box loss and classification loss, which contribute to its detection accuracy and spatial precision.
(g) Conv Module: This module, composed of convolutional layers, BatchNorm, and SiLU activation, standardizes features between layers. It enhances training stability and convergence by preventing issues like gradient vanishing or explosion.
(h) AA2_SPPF Module: The AA2_SPPF module captures global context through multiple MaxPool2d and AdaptiveAvgPool2d operations. This design retains local detail while expanding global feature context, improving the model’s ability to detect expressions at various scales. By reducing the sensitivity to scale variations, AA2_SPPF enhances the accuracy under diverse conditions.

#### 2.2.1. YOLOv8n Model

The YOLOv8n model [8] was chosen as the foundational framework for this study due to its single-stage, regression-based architecture, which enables simultaneous object detection and classification in a single forward pass through the convolutional neural network. This streamlined structure allows for faster inference compared to two-stage algorithms, such as Mask R-CNN [9], which rely on a separate region proposal network. As a result, YOLOv8n is particularly well-suited for real-time applications. Unlike traditional image classification networks like VGG16 and ResNet, which are primarily optimized for classification tasks, YOLOv8n is designed specifically for real-time object detection and handles both classification and localization tasks within the same framework. This capability to multitask allows YOLOv8n to effectively detect and localize facial features, which is crucial for applications like facial expression recognition where spatial accuracy is essential. After evaluating multiple factors, YOLOv8n was selected as the baseline model for sheep facial expression recognition due to its efficient inference and relatively low computational requirements, making it suitable for resource-constrained environments.

**Figure 6:**
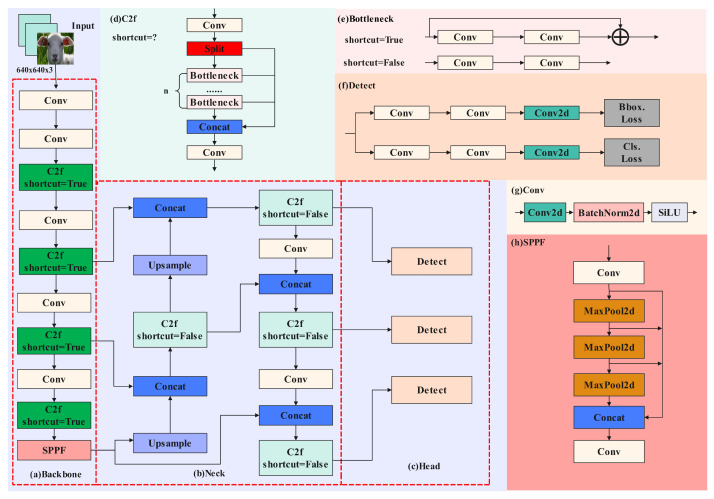
Diagram of the YOLOv8n model architecture.

YOLOv8n offers five model variants—n, s, m, l, and x—each differing in network complexity and size. For this study, the smallest variant, YOLOv8n, was chosen to achieve a balance between computational efficiency and detection accuracy. This choice ensures the model remains manageable in terms of computational requirements, making it adaptable for potential applications in resource-constrained environments, such as mobile and embedded devices, in future research. The YOLOv8n model architecture includes four main components—the input section, Backbone, Neck, and Head—as shown in Figure 6. The roles of each component, corresponding to elements (a) through (g) in the figure, are briefly described as follows, forming the backbone of YOLOv8n’s detection capability:

(a) Input: Standardizes and preprocesses sheep facial expression images for consistent processing across subsequent layers.
(b) Backbone: Extracts essential features from input images, progressively capturing multi-scale features through convolutional layers and C2f modules, serving as the core feature extraction component.
(c) Neck: Integrates and fuses the features at multiple scales through bidirectional mechanisms, utilizing a top-down feature pyramid network (FPN) and a bottom-up path aggregation network (PAN) [10]. This multi-scale fusion enhances both the semantic depth and localization precision of facial expression features.
(d) Head: Outputs the final classification and localization results, with classification and regression tasks refining the fused features to deliver accurate detection outputs.
(e–g) Additional Modules: Components such as the Conv and Bottleneck modules contribute to feature standardization, computational efficiency, and effective information flow throughout the network.

#### 2.2.2. SimAM Module

**Figure 7:**
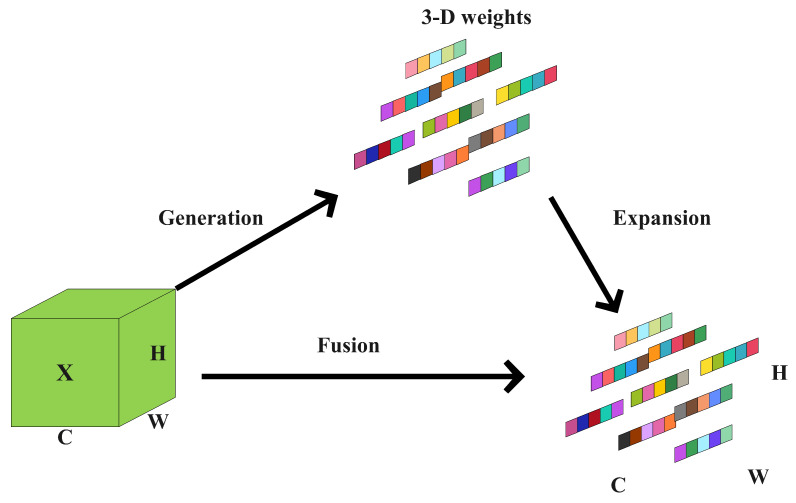
Architecture of the SimAM Attention module.

In difficult environmental conditions, such as low lighting and occlusion, extracting key features from sheep facial expressions can become less effective, reducing the accuracy of recognition. To address these issues, this study integrates the SimAM (Simple Attention Module), a parameter-free attention mechanism, into the baseline model. The addition of SimAM improves the model’s ability to focus on crucial regions of sheep facial expressions, filtering out unimportant factors and enhancing both feature extraction and fusion processes. SimAM, developed based on neuroscience principles, was introduced by Yang et al. as an attention module utilizing 3D weights [11]. It evaluates the significance of individual neurons through spatial inhibition without adding parameters, automatically assigning attention to spatial and channel features. This mechanism improves feature fusion, as shown in Figure 7. The main idea behind SimAM is to build an optimized energy function to highlight the most impactful features for the final prediction. This energy function takes into account the similarities and differences between feature maps and dynamically adjusts channel weights, allowing the model to concentrate on important details while downplaying irrelevant ones [12]. The energy function $e_{t}$ is formulated in Equation (1) as follows:

(1) $$e_{t}\left(w_{t},b_{t},y,x_{i}\right)=\frac{1}{M-1}\sum_{i=1}^{M-1}{\left(-1-(w_{t}x_{i}+b_{t})\right)}^{2}+{\left(-1-(w_{t}t+b_{t})\right)}^{2}+\lambda w_{t}^{2}$$

In this method, the index *i* refers to the spatial dimension, while *M* represents the total number of neurons involved in the network. The target neuron receiving the input feature is designated as *t*, and λ stands for the weight constant. $x_{i}$ represents other neurons in the network. The binary label is denoted by *y*, and *c* is used to indicate whether a neuron is critical within the network.

The weight $w_{t}$ and biase $b_{t}$ were determined using Equation (1) as follows:

(2) $$w_{t}=-\frac{2(t-\mu_{t})}{{\left(t-\mu_{t}\right)}^{2}+2\sigma_{t}^{2}+2\lambda }$$

(3) $$b_{t}=-\frac{1}{2}\left(t+\mu_{t}\right)w_{t}$$

In this context, $\mu_{t}$ and $\sigma_{t}$ represent the mean and variance, respectively, of all neurons within the channel, except for the target neuron t.

The minimum energy function $e_{t}^{*}$ is subsequently derived from Equations (2) and (3), ensuring that the computed values optimize the energy distribution throughout the network as follows:

(4) $$e_{t}^{*}=\frac{4({\hat{\sigma }}^{2}+\lambda )}{{\left(t-\hat{\mu }\right)}^{2}+2{\hat{\sigma }}^{2}+2\lambda }$$

As stated in Equation (4), the energy level differences between neurons follow an inverse proportionality, which suggests that a neuron’s importance can be represented as 1/$e_{t}^{*}$.

As depicted in Figure 7 [11], this relationship facilitates the generation of the fused output map $\tilde{X}$ as follows:

(5) $$\tilde{X}=sigmoid\left(\frac{1}{E}\right)⊙X$$

In this context, E represents the collection of minimal energy functions corresponding to each neuron, while *X* refers to the input feature map.

#### 2.2.3. MobileViTAttention Module

To better capture the global features of small target sheep facial expressions, this study integrates a MobileViT block directly after the second C2f module within the YOLOv8n neck network, linking it to the Detect layer. This adjustment allows for a deeper comprehension of the overall sheep facial expression, enhancing the contextual richness. Consequently, the model’s ability to extract and merge features is significantly improved, especially in challenging conditions like low-light environments.

To meet the demand for lightweight, low-latency, and highly accurate models in mobile visual tasks, Mehta and Rastegari proposed a lightweight backbone network named MobileViT. This network combines the MobileNetV2 structure from convolutional neural networks (CNNs) with the attention mechanism from the Vision Transformer [13,14]. By utilizing fewer channels and adopting a shallower network architecture, this design strikes a balance between performance and efficiency. The MobileViT framework [15] comprises Conv modules, MV2 (MobileNetV2) modules, MobileViT blocks, a global pooling layer, and fully connected layers. At the heart of this network is the MobileViT block, which improves global processing by introducing deeper layers to replace the standard convolution’s local operations. This design maintains the localized feature extraction of convolution but also integrates the ability to capture global context. As a result, MobileViT effectively models both the global and local aspects of sheep facial expressions with fewer parameters, making it a more lightweight solution.

**Figure 8:**
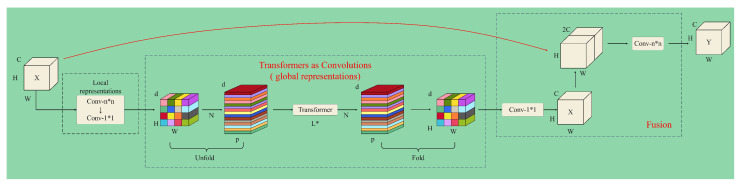
Architecture of the MobileViTAttention module.

The MobileViT block [16], as shown in Figure 8, consists of three key parts: a convolutional module for extracting local features of sheep facial expressions, a Transformer module for capturing global features, and a fusion module for combining both. First, the input feature map of sheep facial expressions undergoes local feature extraction using an n × n convolutional kernel, followed by a 1 × 1 convolution to modify the channel dimensions. The feature map is then passed through a series of Unfold, Transformer, and Fold operations. In this process, Unfold expands the feature map into N flattened, non-overlapping blocks, while the Transformer encodes local and global information together. The Fold operation recombines the encoded blocks [17,18]. Afterward, another 1 × 1 convolution adjusts the channel count, and the enhanced feature map is concatenated with the original input. This is followed by additional convolutional layers to achieve feature fusion. This entire process strengthens feature modeling and representation, thereby boosting model performance.

#### 2.2.4. EfficiCIoU-Loss

To more effectively capture sheep facial expressions that may be obscured by partial occlusion, improve bounding box localization accuracy, accelerate model convergence, and enhance the model’s image learning capabilities, the EfficiCIoU loss function [19] was introduced to replace the original CIoU loss. Serving as the key optimization goal during training, the loss function significantly influences the model’s overall performance. YOLOv8n employs loss functions primarily for classification and bounding box regression, the latter being critical for object localization. High-precision bounding boxes not only improve detection accuracy and robustness but also significantly boost the model’s ability to recognize sheep facial expressions in challenging conditions. Accurate localization minimizes cumulative errors in feature representation, especially for boundary details, which is critical for detecting subtle expression changes even when occlusions or deformations are present. Additionally, precise bounding boxes more effectively isolate the facial region of sheep, reducing background noise and increasing the model’s sensitivity to subtle variations in expression. This is particularly beneficial for sheep facial expression recognition, where minor changes often occur within specific detailed boundary regions. By enhancing bounding box accuracy, the model provides more representative inputs to the feature extraction network, leading to improved overall performance [20].

Bounding box regression losses are typically divided into two categories as follows: $\ell_{n}$-norm-based losses and IoU-based losses. Traditional IoU loss functions focus only on the overlap between predicted and actual boxes, which poses challenges in non-overlapping cases, hindering gradient calculations. To address this, several IoU-based alternatives have been developed, including GIoU [21], DIoU [22], EIoU [23], and CIoU [24]. These loss functions incorporate spatial differences, enhancing localization precision. In YOLOv8n, the bounding box regression loss includes Distribution Focal Loss and CIoU_Loss. CIoU (Complete Intersection over Union) is recognized as one of the most advanced regression losses, factoring in three key geometric elements: overlap area, the distance between center points, and aspect ratio [24]. Rather than solely depending on the overlap measure, CIoU provides a more comprehensive evaluation of box matching by also considering the relationship between the intersection and the union of predicted and ground truth boxes. Additionally, the inclusion of the distance between the center points of these boxes enhances the accuracy of object detection models. Moreover, by taking into account the aspect ratio differences, CIoU allows for more precise adjustments to the shape of the predicted box, which in turn, helps the model better interpret the morphology of the target. The formula for calculating the CIoU loss is as follows:

(6) $$\mathrm{CIoU}\_\mathrm{Loss}=1-\mathrm{CIoU}=1-\mathrm{IoU}+\frac{\rho^{2}\left(b,b^{gt}\right)}{c^{2}}+\alpha v$$

(7) $$\alpha =\frac{v}{1-\mathrm{IoU}+v}$$

(8) $$v=\frac{4}{\pi^{2}}{\left(\arctan\frac{w^{gt}}{h^{gt}}-\arctan\frac{w}{h}\right)}^{2}$$

During the process of bounding box regression, the CIoU loss function faces certain challenges. In particular, it is difficult to adjust both the width and height of the predicted box simultaneously in a straightforward way, as this method does not capture the true variation patterns or the related confidence levels. As a result, when the loss function converges on the ratio between the predicted box and the ground truth box, it can sometimes slow down the model’s optimization. To address this problem, the EIoU_Loss function introduces a breakdown of the aspect ratio influence factor αv within CIoU_Loss. This enables a more accurate calculation of the dimensions of both the predicted and ground truth boxes, effectively overcoming the limitations present in CIoU_Loss [23]. The corresponding formula is as follows:

(9) $$\mathrm{EIoU}\_\mathrm{Loss}=1-\mathrm{EIoU}=1-\mathrm{IoU}+\frac{\rho^{2}\left(b,b^{gt}\right)}{c^{2}}+\frac{\rho^{2}\left(w,w^{gt}\right)}{C_{w}^{2}}+\frac{\rho^{2}\left(h,h^{gt}\right)}{C_{h}^{2}}$$

To enhance both the speed of bounding box adjustments and regression accuracy, the EfficiCIoU_Loss function integrates the benefits of CIoU_Loss and EIoU_Loss. Initially, CIoU_Loss is applied to refine the predicted box’s aspect ratio until it converges to an optimal range. Afterward, EIoU_Loss is employed to make precise edge corrections, ensuring that the width and height achieve the correct proportions [25]. The formula for calculating EfficiCIoU_Loss is shown below.

(10) $$\mathrm{EfficiCIoU}\_\mathrm{Loss}=1-\mathrm{IoU}+\alpha v+\frac{\rho^{2}\left(b^{gt},b\right)}{c^{2}}+\frac{\rho^{2}\left(h^{gt},h\right)}{c_{h}^{2}}+\frac{\rho^{2}\left(w^{gt},w\right)}{c_{w}^{2}}$$

#### 2.2.5. The AA2_SPPF Module

**Figure 9:**
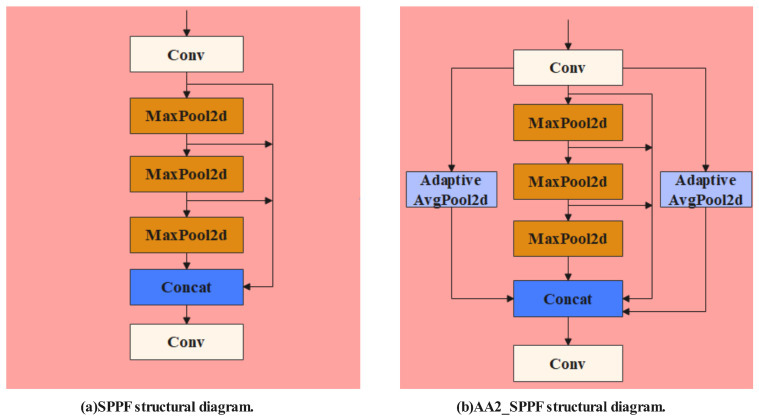
Architecture of the improved SPPF module: (**a**) SPPF structural diagram. (**b**) AA2_SPPF structural diagram.

To reduce computational load and improve processing efficiency, YOLOv8n adopts the Spatial Pyramid Pooling Fusion (SPPF) method, which was initially introduced in YOLOv5 version 6.0. Unlike the original Spatial Pyramid Pooling (SPP), which used multiple convolutional filters, SPPF simplifies this by using a single 5 × 5 convolutional filter along with three max-pooling steps [26]. This structure, as shown in Figure 9a, captures multi-scale features and expands the receptive field, an advantage particularly useful for analyzing sheep facial expressions. However, SPPF primarily focuses on edge information, potentially neglecting background content in images. Since sheep facial expressions often involve subtle changes across different facial regions, especially under challenging conditions like partial occlusion, capturing minor variations in both background and boundary areas is crucial for accurate recognition. To address this, an improved version of the SPPF module, named AA2_SPPF, is proposed in this study, as shown in Figure 9b. The light blue boxes in Figure 9b represent the Adaptive AvgPool2d layers. These layers perform average pooling across various regions, which helps capture fine details and incorporate background information into the feature extraction process, thereby enhancing the model’s robustness. By integrating these Adaptive AvgPool2d layers, the AA2_SPPF module enables more comprehensive feature extraction that considers both global background context and edge details, making it particularly effective for the nuanced task of sheep facial expression recognition. This design reduces noise and improves the model’s sensitivity to subtle expression changes, allowing it to more accurately distinguish between different expressions, even in complex conditions.

## 3. Results

### 3.1. Training Environment Configuration

The experimental setup utilized a Linux Ubuntu 18.04 LTS system, supported by a 12-core Intel Xeon Platinum 8255C CPU operating at 2.50 GHz. Additionally, an NVIDIA GeForce RTX 3080 GPU with 10 GB of VRAM was employed for handling computationally intensive tasks. CUDA 11.0 was used to accelerate these computations, with PyTorch 1.7.0 as the chosen framework for deep learning processes. Python 3.8 was installed in the environment. The model was trained with a batch size of 8 over 150 epochs, starting with a learning rate of 0.01. Momentum was set at 0.937, and the weight decay parameter was 0.0005. The optimizer for this training process was Stochastic Gradient Descent (SGD).

### 3.2. Performance Indicators

To accurately and fairly assess the performance and efficiency of the sheep facial expression recognition model, we employ key evaluation metrics including Precision (P), Recall (R), Mean Average Precision (mAP) and F1-score.

(1) Precision (P) determines how often the model’s positive predictions are correct. The formula for calculating Precision is as follows:

(11) $$P=\frac{TP}{TP+FP}$$

In this analysis, “TP” (True Positives) denotes the count of instances where the model correctly recognizes samples as belonging to the positive class. On the other hand, “FP” (False Positives) signifies the cases where negative samples are mistakenly flagged by the model as positive. Similarly, “FN” (False Negatives) refers to instances where the model incorrectly labels positive samples as negative.

(2) The recall rate (R) measures the fraction of true positive instances among all actual positives. It is a metric that assesses the model’s ability to detect all true positive cases. The formula for recall is as follows:

(12) $$R=\frac{TP}{TP+FN}$$

(3) The Mean Average Precision (mAP) acts as an important metric to evaluate the model’s accuracy over different categories. It is typically assessed at several Intersection over Union (IoU) levels, including 0.5 and a range from 0.5 to 0.95 to provide a more detailed view of the model’s detection performance. The Average Precision (AP) is calculated by determining the area under the Precision-Recall (PR) curve, where precision is represented on the x-axis and recall is on the y-axis. The mAP is then derived by taking the average of the AP values across all categories. Below is the relevant formula:

(13) $$mAP=\frac{1}{c}\sum_{i=1}^{c}AP_{i}$$

(4) F1-score is a critical metric for evaluating model performance, particularly when dealing with imbalanced datasets. It provides a harmonic mean of precision and recall, offering a single measure that balances both the ability to correctly identify positive instances and the ability to avoid false positives. The formula for F1-score is as follows:

(14) $$F1-score=2\cdot \frac{P\cdot R}{P+R}$$

### 3.3. SMEA-YOLOv8n Model Results for Sheep Facial Expression Recognition

#### 3.3.1. Comparative Analysis of Attention Mechanisms

To assess the role of the SimAM module in improving sheep facial expression recognition under difficult conditions like poor lighting, we used the YOLOv8n model as our baseline. The SimAM module, which does not require any parameters, was inserted at multiple points in the network: within the SPPF module of the Backbone, the C2f module linked to the Detect component in the Neck, and another C2f module in the Neck itself. These positions were labeled as After_SPPF, Concat_Detect, and Neck_C2f, respectively. As seen in Table 3, adding SimAM to the Backbone’s SPPF module yielded the weakest results, with mAP and Precision dropping by 0.4% and 3.5%, respectively, compared to the baseline. In contrast, placing SimAM in the C2f module linked to the Detect section of the Neck network produced the highest gains, with an mAP of 90.3%, which is 2.3% higher than the baseline, and a Recall of 82.5%. Notably, there was no increase in GFLOPs, meaning the model’s complexity stayed the same. To provide a balanced comparison of how different attention modules impacted performance, we also tested SimAM alongside three other mechanisms: coordAtt (CA) [27], CBAM [28], and CReToNeXt [29]. These were integrated into the same spots in the Backbone and Neck networks of YOLOv8n while ensuring the rest of the network was unchanged to maintain consistent experimental conditions. According to Table 4, SimAM outperformed coordAtt, CBAM, and CReToNeXt, showing mAP improvements of 1.8%, 7.5%, and 1.2%, respectively. Additionally, SimAM had a lower computational cost per sheep face image, measured in GFLOPs, demonstrating its effectiveness in focusing on small features and enhancing recognition accuracy.

To better capture contextual details in challenging conditions like poor lighting, this study improves the baseline model by introducing the SimAM attention module, paired with the MobileViT block, which utilizes Transformer architecture. This combination allows the model to grasp both the broad and detailed features of sheep facial expressions, while still keeping the parameter count low, aiding in building a lightweight model. The effect of integrating MobileViT’s attention mechanism was tested by replacing SimAM modules in the C2f layers connected to the Detect module of the YOLOv8n_SimAM model. This replacement created three versions: C2f_1, C2f_2, and C2f_3. As shown in Table 5, C2f_2 had the best results in recognizing facial expressions. Although there was a small rise in computational load, measured in GFLOPs, the model showed improvements in mAP, Precision, and Recall, reaching 90.6%, 83.9%, and 85.8%, respectively, which represent increases of 0.3%, 1.0%, and 3.3% compared to the original YOLOv8n_SimAM model.

#### 3.3.2. Impact of Loss Function Modifications: Comparative Analysis

To evaluate the performance of the EfficiCIoU loss function in accelerating model convergence, this study incorporates the SimAM module and MobileViTAttention into the base model. A comparison was made between the EfficiCIoU loss function and other commonly used loss functions, such as CIoU, EIoU, XIoU [30], WIoU [31], and SIoU [32]. As shown in Table 6, the model employing the EfficiCIoU loss function outperforms the alternatives in terms of mAP, Precision, and Recall. Specifically, mAP improved by 1.5%, Precision stayed consistent, and Recall increased by 3.7%, resulting in more accurate sheep face identification and greater confidence in recognizing sheep facial expressions.

#### 3.3.3. Performance Impact of SPPF Module Modifications

To further validate the effectiveness of the AA2_SPPF enhancement, we conducted a comparative analysis against the AM_SPPF module. This analysis involved integrating both global average pooling and global max pooling layers into the SPPF module. As outlined in Table 7, the AA2_SPPF module exhibited clear performance improvements, with increases of 4.6%, 7.6%, and 2.5% in mAP, Precision, and Recall, respectively, compared to the AM_SPPF module. Additionally, it also outperformed the original SPPF module, showing gains of 0.4%, 2.5%, and 1.5% in the same metrics. These results highlight that the AA2_SPPF enhancement substantially enhances the network’s capacity to capture detailed and small-scale sheep facial expression features.

### 3.4. Ablation Experiment

**Figure 10:**
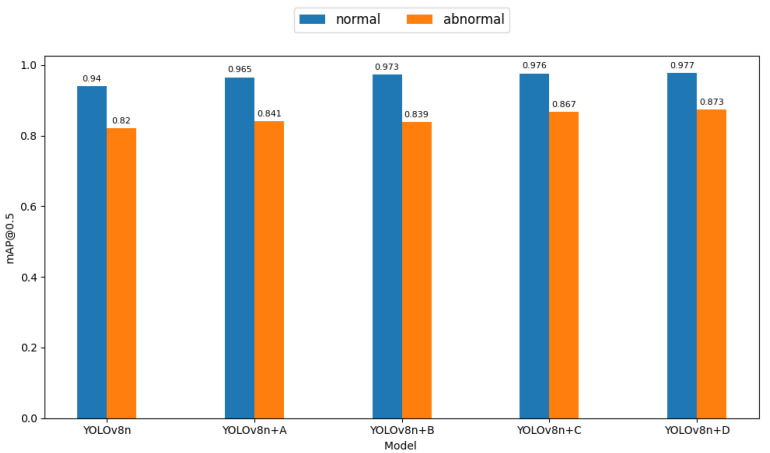
Comparative mAP@0.5 evaluation of enhanced sheep facial expression models.

To comprehensively evaluate how the proposed algorithmic modifications affect model performance, a series of ablation studies were carried out. These involved step-by-step adjustments to the YOLOv8n model. First, the SimAM attention module was added to three C2f modules linked to the detection head within the neck network. Then, in the third C2f module, the SimAM attention was swapped for the MobileViTAttention module. Additionally, the loss function was updated to EfficiCIoU. Lastly, the SPPF module in the backbone was refined to AA2_SPPF. As shown in Table 8, the proposed SMEA-YOLOv8n model achieved a mean average precision (mAP) of 92.5% for sheep facial expression recognition, representing a 4.5% increase over the baseline YOLOv8n model. The recall improved by 9.1%, precision increased by 2.8%, and the F1-score improved by 6.0%, all of which significantly lowered false detections. Table 9 and Figure 10 further demonstrate the performance improvements of the enhanced YOLOv8n model across normal and abnormal expressions. For the baseline YOLOv8n model, the mAP@0.5 for normal expressions reaches 94.0%, which is substantially higher compared to 82.0% for abnormal expressions, reflecting the impact of the dataset imbalance. With the addition of the SimAM module, the mAP@0.5 increases by 2.5% for normal expressions and 2.1% for abnormal expressions, with recall for abnormal expressions improving slightly (+1.4%). The MobileViTAttention module brings a 3.3% increase in mAP@0.5 for normal expressions and a 1.9% increase for abnormal expressions, alongside a substantial recall improvement for abnormal expressions (+8.7%), showcasing its ability to enhance feature extraction and context understanding. The EfficiCIoU loss function brings a more pronounced improvement for abnormal expressions, with a 4.7% increase in mAP@0.5 and a significant recall boost (+11.0%), despite a minor precision decrease (−2.1%). Finally, replacing the SPPF module with AA2_SPPF results in the largest improvement for abnormal expressions, with a 5.3% increase in mAP@0.5, alongside a notable 15.0% increase in recall, highlighting its ability to mitigate class imbalance effectively. Overall, these enhancements demonstrate consistent improvements across both expression categories, with the abnormal class benefitting the most in terms of recall, thereby addressing the challenges posed by class imbalance in sheep facial expression recognition.

**Figure 11:**
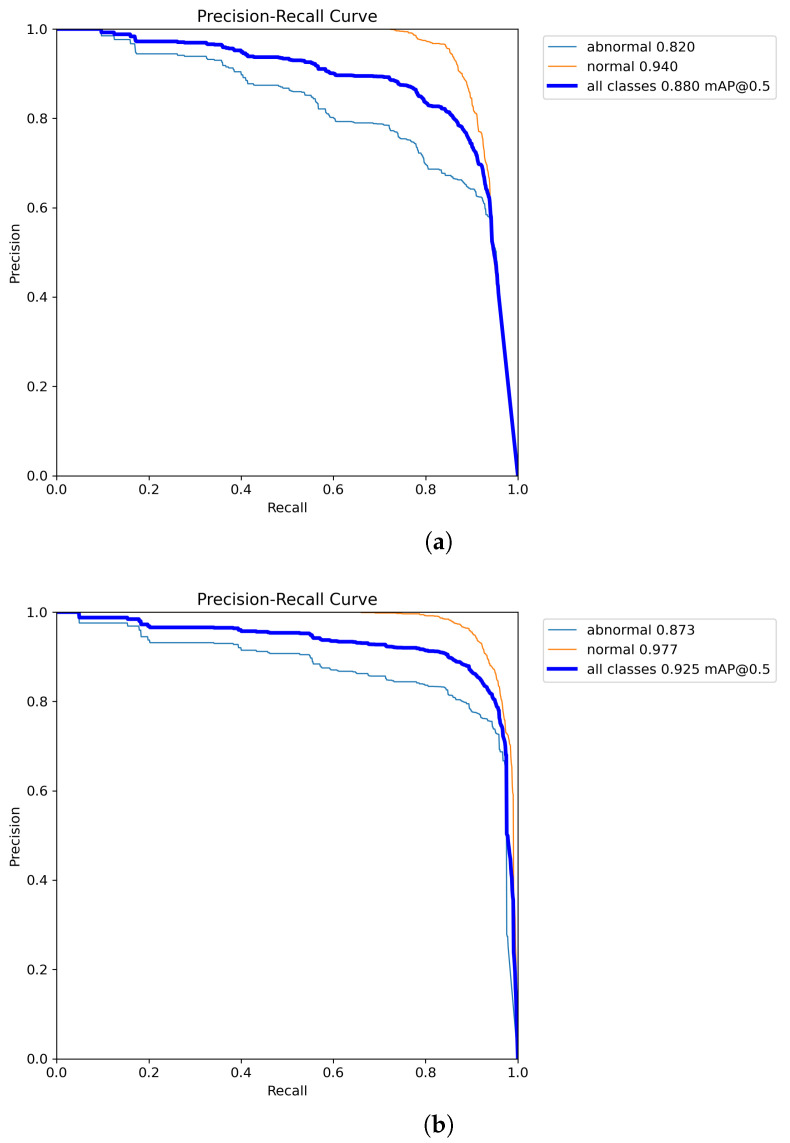
Comparison of P-R curves before and after model enhancement. (**a**) P-R curve in YOLOv8n. (**b**) P-R curve in SMEA-YOLOv8n.

**Figure 12:**
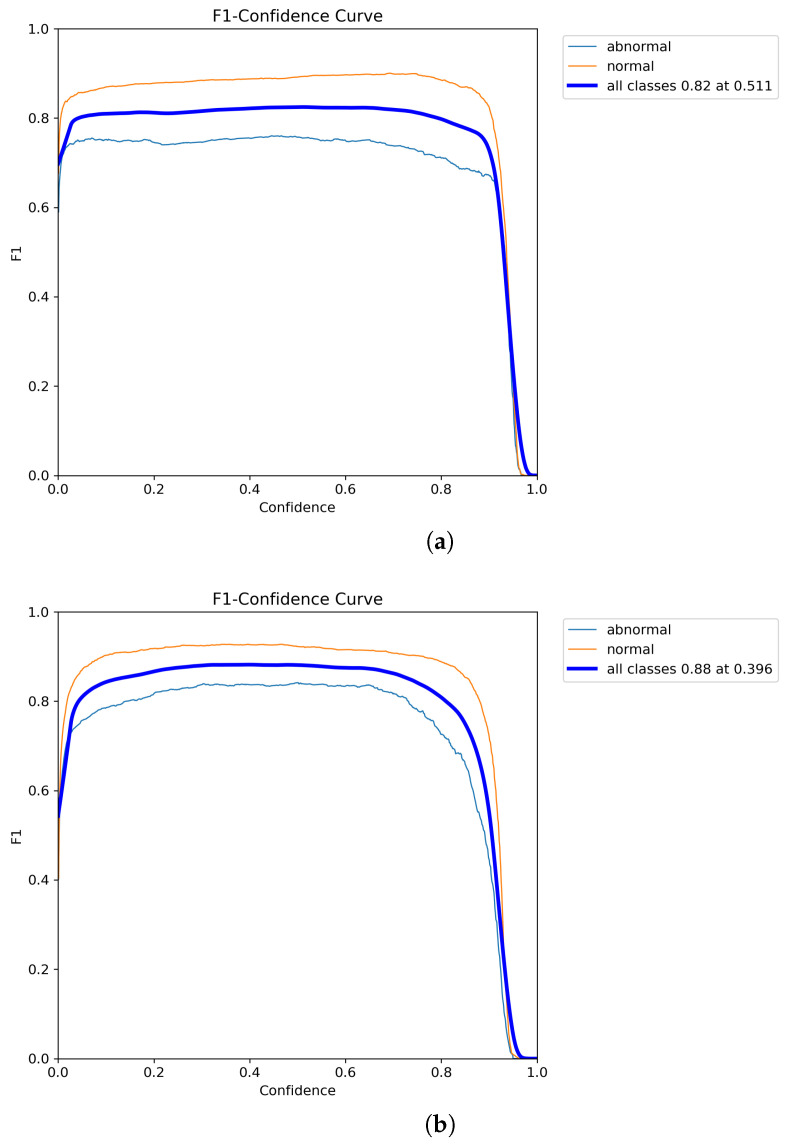
Comparison of F1-score curves before and after model enhancement. (**a**) F1-score curve in YOLOv8n. (**b**) F1-score curve in SMEA-YOLOv8n.

**Figure 13:**
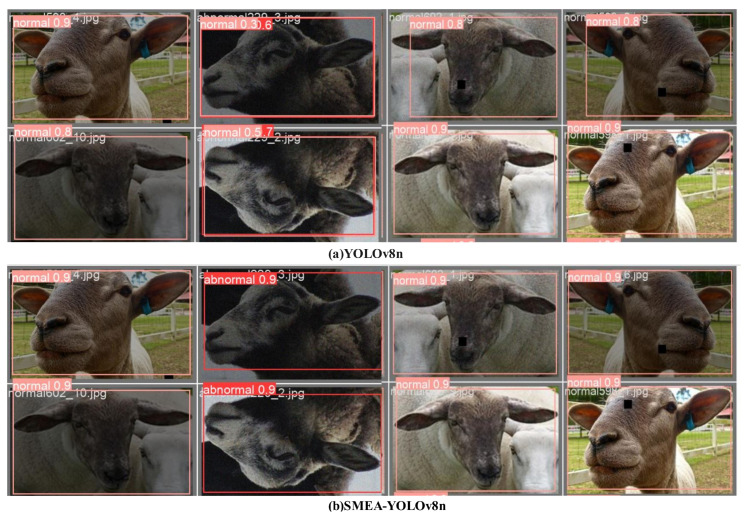
Visual comparison of sheep facial expression recognition pre- and post-YOLOv8n model enhancements.

To better highlight the improvements introduced by the proposed model, the Precision-Recall (P-R) curves for the YOLOv8n model, before and after the enhancements, are shown in Figure 11. As the number of iterations increases, the enhanced model, SMEA-YOLOv8n, consistently achieves better Precision, Recall, and mAP values than the baseline YOLOv8n. These findings indicate that the adjustments to the model significantly improve the accuracy of sheep facial expression recognition and effectively reduce false detections. In this study, the dataset is imbalanced, with a significantly higher number of normal sheep facial expression samples compared to abnormal ones. This skew can lead the model to prioritize learning features of normal expressions, potentially compromising its ability to detect abnormal expressions and increasing the risk of false negatives. As shown in Figure 12a, the F1-score curve of the YOLOv8n model demonstrates a high score for the normal category, which remains relatively stable across most confidence intervals, indicating satisfactory performance in detecting normal expressions. However, the F1-score curve for the abnormal category is generally low and exhibits a marked decline as confidence increases, highlighting the model’s insufficient detection capability for abnormal expressions. Consequently, the overall highest F1-score for the YOLOv8n model is 82.0% across all categories, with an optimal confidence threshold of 0.511 for the best sheep facial expression detection performance. In contrast, the improved SMEA-YOLOv8n model exhibits a more balanced detection performance, as depicted in Figure 12b. The F1-score for the normal category is comparable to that of YOLOv8n, indicating that the improvements do not negatively affect the detection of normal expressions. Notably, the F1-score curve for the abnormal category in SMEA-YOLOv8n shows significant improvement, with higher scores in the medium and low confidence intervals, and remains relatively stable as confidence increases. This suggests enhanced sensitivity and robustness in detecting abnormal expressions. The SMEA-YOLOv8n model achieves the highest F1-score of 88.0% across all categories, representing a 6.0% increase compared to YOLOv8n, and achieves its optimal detection performance at a lower confidence threshold of 0.396. This indicates that the improved model can achieve high recognition accuracy at a lower confidence level, which helps reduce the risk of missing abnormal expressions. Moreover, a visual comparison of YOLOv8n and SMEA-YOLOv8n, presented in Figure 13, further supports these results. The YOLOv8n model shows some issues with false detections and lower accuracy, whereas the SMEA-YOLOv8n model displays noticeable improvements in recognition accuracy, addressing both false detection and precision challenges. This confirms the effectiveness of the algorithmic enhancements.

### 3.5. Comparative Analysis of Different Models for Sheep Facial Expression Recognition

The effectiveness of the SMEA-YOLOv8n algorithm was evaluated through comparative experiments with leading object detection models. These experiments were conducted using consistent datasets, experimental settings, and training parameters. The results are summarized in Table 10. Due to its complex architecture and large model size, Faster-RCNN is less suitable for sheep face expression recognition tasks. In contrast, the SMEA-YOLOv8n model outperforms YOLOv5s and YOLOv7, achieving improvements of 8.2% and 5.7% in mAP@0.5, respectively, and increases in precision of 11.3% and 6.5%. Additionally, recall rates improved by 10.6% and 11.0%, respectively. The model size of SMEA-YOLOv8n is 8.3 MB, representing a reduction of 6.1 MB compared to YOLOv5s and a significant decrease of 66.5 MB compared to YOLOv7. These results demonstrate that the SMEA-YOLOv8n algorithm offers higher efficiency and greater accuracy for sheep face expression recognition tasks.

## 4. Discussion

**Figure 14:**
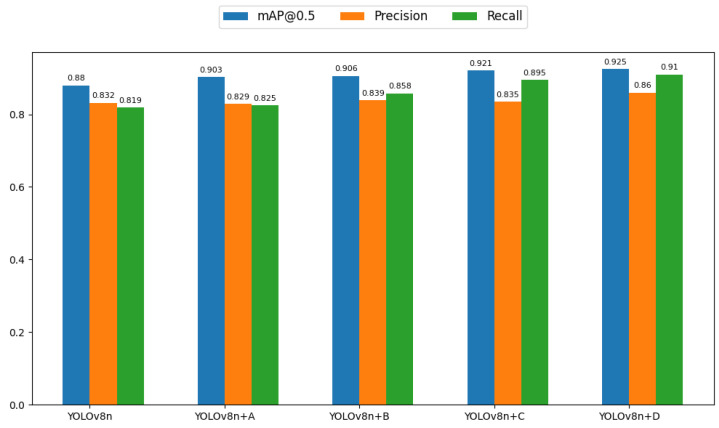
Comparative analysis of mAP@0.5, Precision, and Recall across various YOLOv8n model enhancements.

As shown in Figure 14, the mAP@0.5 scores of the modified YOLOv8n models demonstrate clear improvements across different setups. Adding the SimAM module to the neck of the network (YOLOv8n+A) led to a noticeable increase in mAP for normal sheep facial expressions, going from 0.94 to 0.965, and for abnormal expressions from 0.82 to 0.841. This change can be credited to SimAM’s ability to focus on important facial areas, especially under challenging conditions such as occlusion, which enhances expression recognition accuracy. Additional improvement was seen when the MobileViTAttention module (YOLOv8n+B) was introduced, increasing the mAP for normal expressions to 0.973, though the gain for abnormal expressions was less significant. The MobileViTAttention module leverages Transformer-based self-attention to capture global information while blending it with local features from convolutional layers, thus boosting the recognition accuracy overall. Replacing the loss function with EfficiCIoU (YOLOv8n+C) led to further improvements, with the mAP for normal expressions reaching 0.976 and for abnormal expressions increasing to 0.867. The EfficiCIoU loss function overcomes the limitations of CIoU and EIoU, improving the model’s ability to adjust prediction boxes and refine regression accuracy, thereby improving recognition performance. Finally, replacing SPPF in the backbone network with AA2_SPPF (YOLOv8n+D) resulted in slight gains, with mAP increasing to 0.977 for normal expressions and 0.873 for abnormal expressions. AA2_SPPF improves feature extraction by utilizing two global average pooling layers, allowing the model to detect more nuanced and essential features of facial expressions. The SMEA-YOLOv8n model significantly improved in detecting sheep facial expressions, with accuracy for normal expressions rising from 0.913 to 0.951 and for abnormal expressions increasing from 0.75 to 0.769. This demonstrates a better identification of both non-painful and painful expressions. Additionally, the mAP score increased from 0.880 to 0.925, reflecting an enhanced ability to recognize these expressions. These improvements in precision, mAP, and recall indicate that the SMEA-YOLOv8n model provides a more accurate and comprehensive understanding of sheep expressions, enabling the quicker detection of abnormal conditions and potentially reducing disease risks caused by late detection, which is beneficial for animal welfare in smart farming systems.

The current dataset is limited by a relatively small number of abnormal sheep facial expressions, leading to a significant class imbalance between normal and abnormal samples. This imbalance may affect the generalizability of the findings. To address this, future work will focus on expanding the dataset by incorporating more samples, particularly those that represent facial expressions associated with common diseases or discomforts, such as foot rot and mastitis. This will improve the dataset’s diversity and ensure more robust model training. Additionally, the use of Generative Adversarial Networks (GANs) is proposed to generate synthetic abnormal facial expression data, which could help alleviate data imbalance and enhance the model’s robustness. However, since the current abnormal facial expression data have not been calibrated with clinically diagnosed conditions, future studies will need to integrate real-world diagnoses, such as foot rot and mastitis, in order to better characterize and validate the abnormal group. Despite the improvements achieved with the updated model, certain areas remain that require further refinement. The inclusion of additional modules has slightly increased both the parameter count and computational complexity, as well as reduced the frame rate (FPS) compared to the original YOLOv8n. To mitigate this, future work will explore alternative convolutional layers to replace standard convolutions, aiming to reduce unnecessary features and lower computational demand, while maintaining or improving the overall performance. Furthermore, optimizing the Detect head for more efficient spatial feature extraction is a priority, as this could reduce redundant computations and memory usage, improving processing efficiency. Finally, future work will focus on developing lightweight models that are optimized for mobile platform deployment. This effort aims to improve the model’s accessibility and broaden its practical applicability, enabling easier implementation in real-world scenarios, particularly in resource-constrained environments.

## 5. Conclusions

This research presents an improved algorithm based on YOLOv8n for recognizing sheep facial expressions, focusing on reducing errors and enhancing accuracy under various environmental conditions. To tackle these issues, the SimAM and MobileViTAttention modules were incorporated into the network’s C2f layer. The SimAM module evaluates neuron significance via spatial inhibition, enabling the model to distribute attention more effectively across spatial and channel features, thus boosting its feature extraction abilities. Simultaneously, the MobileViT module captures both global and local information on sheep facial expressions with fewer parameters. Moreover, the loss function was replaced with the EfficiCIoU loss function, which improves the speed and accuracy of bounding box regression by refining the aspect ratio using CIoU_Loss, and adjusting the edges through EIoU_Loss until the optimal width-to-height ratio is achieved. This approach leads to better results in recognizing sheep facial expressions. Additionally, the upgraded AA2_SPPF module captures global context information, reducing the effect of varying scales and allowing the network to extract vital details from small and nuanced expressions. Experimental findings show that, compared to the baseline YOLOv8n model, the SMEA-YOLOv8n model provides improvements of 4.5%, 2.8%, 9.1%, and 6.0% in mAP, Precision, Recall, and F1-score, respectively. Specifically, normal sheep facial expressions saw a 3.7% increase in mAP, while abnormal (painful) expressions showed a 5.3% improvement.

## Figures and Tables

**Table 1:** The Sheep Pain Facial Expression Scale (SPFES).

Eye Constriction Status	Ear Rotation Degree	Nostril Configuration	Assessment Score
Eyes fully open	Ears fully visible	U-shaped nostrils	0 point
			(No indication of pain)
Eyes half-closed	Ears partially visible	Slightly V-shaped nostrils	1 point
			(Potential pain present)
Eyes nearly fully closed	Ears not visible	Distinct V-shaped nostrils	2 points
			(High likelihood of pain)

**Table 2:** Results of newly incorporated sheep facial expression image scoring.

Evaluation Score (Points)	0	1	2	3	4	5	6	7	8	9	10
**Number of images**	596	93	12	5	23	8	14	0	0	0	0

**Table 3:** Comparison of SimAM addition to YOLOv8n model at different positions.

Method	mAP@0.5 (%)	Precision (%)	Recall (%)	GFLOPs
baseline	88.0	83.2	81.9	8.1
After_SPPF	87.6	79.7	88.6	8.1
Concat_Detect	90.3	82.9	82.5	8.1
Neck_C2f	88.7	82.5	89.4	8.1

**Table 4:** Comparison of models incorporating other attention mechanisms.

Method	mAP@0.5 (%)	Precision (%)	Recall (%)	GFLOPs
baseline	88.0	83.2	81.9	8.1
CA	88.5	83.9	84.4	8.2
CBAM	82.8	82.1	82.7	8.9
CReToNeXt	89.1	84.2	87.5	9.2
SimAM	90.3	82.9	82.5	8.1

**Table 5:** Comparison of MobileViTAttention addition to YOLOv8n model at different positions.

Method	mAP@0.5 (%)	Precision (%)	Recall (%)	GFLOPs
YOLOv8n_SimAM	90.3	82.9	82.5	8.1
C2f_1	88.7	82.4	88.6	11.1
C2f_2	90.6	83.9	85.8	11.1
C2f_3	89.5	81.6	87.0	11.1

**Table 6:** Comparison of loss function.

Method	mAP@0.5 (%)	Precision (%)	Recall (%)
CIoU	90.6	83.9	85.8
EIoU	88.7	82.6	85.4
XIoU	89.8	83.2	84.1
WIoU	85.3	79.1	84.8
SIoU	85.8	80.3	79.4
EfficiCIoU	92.1	83.5	89.5

**Table 7:** Comparison of different SPPF improved modules.

Method	mAP@0.5 (%)	Precision (%)	Recall (%)
SPPF	92.1	83.5	89.5
AM_SPPF	87.9	78.4	88.5
AA2_SPPF	92.5	86.0	91.0

**Table 8:** Ablation experiment.

SimAM	MobileViTAttention	EfficiCIoU	AA2_SPPF	mAP@0.5 (%)	Precision (%)	Recall (%)	F1-Score (%)	Model Size (MB)
✗	✗	✗	✗	88.0	83.2	81.9	82.0	6.2
✓	✗	✗	✗	90.3	82.9	82.5	82.0	6.2
✓	✓	✗	✗	90.6	83.9	85.8	84.0	8.2
✓	✓	✓	✗	92.1	83.5	89.5	85.0	8.2
✓	✓	✓	✓	92.5	86.0	91.0	88.0	8.3

**Table 9:** Comparison of sheep facial expression recognition.

Method	Normal			Abnormal		
	mAP@0.5 (%)	Precision (%)	Recall (%)	mAP@0.5 (%)	Precision (%)	Recall (%)
YOLOv8n	94.0	91.3	87.1	82.0	75.0	76.7
+SimAM	96.5 (+2.5)	90.9	86.9	84.1 (+2.1)	74.9	78.1 (+1.4)
+MobileViT	97.3 (+3.3)	93.5 (+2.2)	86.2	83.9 (+1.9)	74.3	85.4 (+8.7)
+EfficiCIoU	97.6 (+3.6)	94.0 (+2.7)	91.4 (+4.3)	86.7 (+4.7)	73.0	87.7 (+11.0)
+AA2_SPPF	97.7 (+3.7)	95.1 (+3.8)	90.3 (+3.2)	87.3 (+5.3)	76.9 (+1.9)	91.7 (+15.0)

**Table 10:** Comparison of different models for sheep facial expression recognition.

Model	mAP@0.5 (%)	Precision (%)	Recall (%)	Model Size (MB)
Faster-RCNN	81.6	74.0	76.4	81.5
YOLOv5s	84.3	74.7	80.4	14.4
YOLOv7	86.8	79.5	80.0	74.8
YOLOv8n	88.0	83.2	81.9	6.2
ours	92.5	86.0	91.0	8.3

## Data Availability

Data available on request due to restrictions.

## References

[1] Yao Z., Tan H., Tian F., Zhou Y., Zhang C. (2021). Research progress of computer vision technology in wisdom sheep farm. China Feed.

[2] McLennan K.M., Rebelo C.J.B., Corke M.J., Holmes M.A., Leach M.C., Constantino-Casas F. (2016). Development of a facial expression scale using footrot and mastitis as models of pain in sheep. Appl. Anim. Behav. Sci..

[3] Lu Y., Mahmoud M., Robinson P. Estimating sheep pain level using facial action unit detection. Proceedings of the 12th IEEE International Conference on Automatic Face & Gesture Recognition (FG 2017).

[4] Pessanha F., McLennan K., Mahmoud M. Towards automatic monitoring of disease progression in sheep: A hierarchical model for sheep facial expressions analysis from video. Proceedings of the 15th IEEE International Conference on Automatic Face and Gesture Recognition (FG 2020).

[5] Noor A., Zhao Y., Koubâa A., Wu L., Khan R., Abdalla F.Y. (2020). Automated sheep facial expression classification using deep transfer learning. Comput. Electron. Agric..

[6] HAN D., WANG B., WANG L., HOU Y., TIAN H., ZHANG S. (2021). Individual Pain Recognition Method of Sheep Based on Improved VGGNet. Trans. Chin. Soc. Agric. Mach..

[7] Jiang B., Li N., Cui X., Zhang Q., Zhang H., Li Z., Liu W. (2024). Research on facial expression recognition algorithm based on improved MobileNetV3. EURASIP J. Image Video Process..

[8] Wang G., Chen Y., An P., Hong H., Hu J., Huang T. (2023). UAV-YOLOv8: A small-object-detection model based on improved YOLOv8 for UAV aerial photography scenarios. Sensors.

[9] Tian Y., Yang G., Wang Z., Li E., Liang Z. (2020). Instance segmentation of apple flowers using the improved mask R–CNN model. Biosyst. Eng..

[10] Guo C., Fan B., Zhang Q., Xiang S., Pan C. Augfpn: Improving multi-scale feature learning for object detection. Proceedings of the IEEE/CVF Conference on Computer Vision and Pattern Recognition.

[11] Yang L., Zhang R., Li L., Xie X. Simam: A simple, parameter-free attention module for convolutional neural networks. Proceedings of the International Conference on Machine Learning.

[12] You H., Lu Y., Tang H. (2023). Plant disease classification and adversarial attack using SimAM-EfficientNet and GP-MI-FGSM. Sustainability.

[13] Mehta S., Rastegari M. (2021). Mobilevit: Light-weight, general-purpose, and mobile-friendly vision transformer. arXiv.

[14] Han K., Wang Y., Chen H., Chen X., Guo J., Liu Z., Tang Y., Xiao A., Xu C., Xu Y. (2022). A survey on vision transformer. IEEE Trans. Pattern Anal. Mach. Intell..

[15] SHEN P., LI X., YANG N., CHEN A. (2024). Lightweight YOLOv8 PCB defect detection algorithm based on triple attention. Microelectron. Comput..

[16] Yi S., Chen M., Liu X., Li J., Chen L. (2023). HAFFseg: RGB-Thermal semantic segmentation network with hybrid adaptive feature fusion strategy. Signal Process. Image Commun..

[17] Wang R., Liu D., Zhang J. (2024). Wear-YOLO:Research on Detection Methods of Safety Equipment for Power Personnel in Substations. J. Comput. Eng. Appl..

[18] Mehta S., Rastegari M. (2022). Separable self-attention for mobile vision transformers. arXiv.

[19] Ji C., Jia X., Huang X., Zhou S., Chen G., Zhu Y. (2024). FusionNet: Detection of Foreign Objects in Transmission Lines During Inclement Weather. IEEE Trans. Instrum. Meas..

[20] Zheng Z., Wang P., Ren D., Liu W., Ye R., Hu Q., Zuo W. (2021). Enhancing Geometric Factors in Model Learning and Inference for Object Detection and Instance Segmentation. IEEE Trans. Cybern..

[21] Rezatofighi H., Tsoi N., Gwak J., Sadeghian A., Reid I., Savarese S. Generalized intersection over union: A metric and a loss for bounding box regression. Proceedings of the IEEE/CVF Conference on Computer Vision and Pattern Recognition.

[22] Zheng Z., Wang P., Liu W., Li J., Ye R., Ren D. Distance-IoU loss: Faster and better learning for bounding box regression. Proceedings of the AAAI Conference on Artificial Intelligence.

[23] Zeng W., Huang J., Wen S., Fu Z. (2023). A masked-face detection algorithm based on M-EIOU loss and improved ConvNeXt. Expert Syst. Appl..

[24] Du S., Zhang B., Zhang P., Xiang P. An improved bounding box regression loss function based on CIOU loss for multi-scale object detection. Proceedings of the 2021 IEEE 2nd International Conference on Pattern Recognition and Machine Learning (PRML).

[25] Qiu C., Tang H., Yang Y., Wan X., Xu X., Lin S., Lin Z., Meng M., Zha C. (2024). Machine vision-based autonomous road hazard avoidance system for self-driving vehicles. Sci. Rep..

[26] Wang X., Gao H., Jia Z., Li Z. (2023). BL-YOLOv8: An improved road defect detection model based on YOLOv8. Sensors.

[27] Zhao X., Zhang W., Zhang H., Zheng C., Ma J., Zhang Z. (2024). ITD-YOLOv8: An Infrared Target Detection Model Based on YOLOv8 for Unmanned Aerial Vehicles. Drones.

[28] Yu H., Wang J., Han Y., Fan B., Zhang C. (2024). Research on an intelligent identification method for wind turbine blade damage based on CBAM-BiFPN-YOLOV8. Processes.

[29] Nie L., Li B., Du Y., Jiao F., Song X., Liu Z. (2024). Deep learning strategies with CReToNeXt-YOLOv5 for advanced pig face emotion detection. Sci. Rep..

[30] Xin Z., Lu T., Li X. Detection of train bottom parts based on XIoU. Proceedings of the 2019 International Conference on Robotics Systems and Vehicle Technology.

[31] Tong Z., Chen Y., Xu Z., Yu R. (2023). Wise-IoU: Bounding box regression loss with dynamic focusing mechanism. arXiv.

[32] Chen Y., Xu H., Chang P., Huang Y., Zhong F., Jia Q., Chen L., Zhong H., Liu S. (2024). CES-YOLOv8: Strawberry Maturity Detection Based on the Improved YOLOv8. Agronomy.

